# Bruxism treatment on Youtube: evaluating reliability and information accuracy

**DOI:** 10.1186/s12903-024-04571-5

**Published:** 2024-07-15

**Authors:** Onur Odabaşı, Güzin Neda Hasanoğlu Erbaşar, Kevser Sancak

**Affiliations:** Department of Oral and Maxillofacial Surgery, Faculty of Dentistry, Ankara Yıldırım Beyazıt University, Ankara, Turkey

**Keywords:** Youtube, Discern, Bruxism treatment, Internet, Patient information

## Abstract

**Background:**

The aim of this study was to evaluate the content and quality of videos about bruxism treatments on YouTube, a platform frequently used by patients today to obtain information.

**Methods:**

A YouTube search was performed using the keywords “bruxism treatment” and “teeth grinding treatment”. “The sort by relevance” filter was used for both search terms and the first 150 videos were saved. A total of 139 videos that met the study criteria were included in the study. Videos were classified as poor, moderate or excellent based on a usefulness score that evaluated content quality. The modified DISCERN tool was also used to evaluate video quality. Additionally, videos were categorized according to the upload source, target audience and video type. The types of treatments mentioned in the videos and the demographic data of the videos were recorded.

**Results:**

According to the usefulness score, 59% of the videos were poor-quality, 36.7% were moderate-quality and 4.3% were excellent-quality. Moderate-quality videos had a higher interaction index than excellent-quality videos (*p* = 0.039). The video duration of excellent-quality videos was longer than that of moderate and poor-quality videos (*p* = 0.024, *p* = 0.002). Videos with poor-quality content were found to have significantly lower DISCERN scores than videos with moderate (*p* < 0.001) and excellent-quality content (*p* = 0.008). Additionally, there was a significantly positive and moderate (*r* = 0.446) relationship between DISCERN scores and content usefulness scores (*p* < 0.001). There was only a weak positive correlation between DISCERN scores and video length (*r* = 0.359; *p* < 0.001). The videos uploaded by physiotherapists had significantly higher views per day and viewing rate than videos uploaded by medical doctors (*p* = 0.037), university-hospital-institute (*p* = 0.024) and dentists (*p* = 0.006). The videos uploaded by physiotherapists had notably higher number of likes and number of comments than videos uploaded by medical doctors (*p* = 0.023; *p* = 0.009, respectively), university-hospital-institute (*p* = 0.003; *p* = 0.008, respectively) and dentists (*p* = 0.002; *p* = 0.002, respectively).

**Conclusions:**

Although the majority of videos on YouTube about bruxism treatments are produced by professionals, most of the videos contain limited information, which may lead patients to debate treatment methods. Health professionals should warn patients against this potentially misleading content and direct them to reliable sources.

**Supplementary Information:**

The online version contains supplementary material available at 10.1186/s12903-024-04571-5.

## Background

Nowadays, an increasing number of users are utilizing the internet to obtain health-related information, [1, 2] and searching for health information online is among the most popular online activities [3] Indeed, a 2012 survey conducted in the United States revealed that 72% of internet users had searched for health information online in the past year, and 35% of them had used the internet as a diagnostic tool [4] Youtube is a video platform that offers visual and audio content on websites [5] and the viewing rate of health-related videos on YouTube is increasing day by day [6] According to the Health Information National Trends Survey (HINTS) conducted by the National Cancer Institute, the proportion of people who watched health-related YouTube videos in the past year increased from 39.7% in 2020 (HINTS 5 Cycle 4) to 59% in the 2022 survey (HINTS 6) [7] A study conducted in 2024 with 3000 YouTube users found that 84.6% of the participants watched health-related videos on YouTube and 84.7% made decisions based on what they watched [8].

YouTube can be used by both professionals and non-professionals [9] Especially for non-professional users, videos that combine text, audio and visuals make health information more understandable and interesting [10] and can provide information on many different topics in the field of health, such as vitamin D use, [11] pediatric scoliosis, [12] covid pandemic, [13] food poisoning, [6] orthognathic surgery. [14] While professionals have the opportunity to filter the information in the videos they watch through the knowledge they already have, medical books and literature, non-professional users are vulnerable in this regard [15] and for these people, the reliability of health-related information on YouTube becomes much more important. For a portion of the health-related videos uploaded to this platform are uploaded by non-health professionals, [6] and they do not undergo any peer review or control mechanism. [5].

Bruxism is characterized by a repetitive muscle masticatory activity accompanied by clenching and grinding of teeth and/or bracing and thrusting of mandible according to international consensus on bruxism. These conditions can also be divided into sleep bruxism and awake bruxism [16] Bruxism can have many consequences such as tooth fracture, headache, temporomandibular dysfunction, and muscle fatique [17].

Bruxism has a multifactorial etiology that includes psychological factors such as stress, anxiety, and depression, genetic predisposition, neurotransmitter imbalances, sleep disorders, the use of tobacco, alcohol, and caffeine, certain medications, Parkinson’s disease, attention deficit hyperactivity disorder (ADHD), gastroesophageal reflux disease and obstructive sleep apnea (OSA). Each of these factors represents potential risk contributors to the development of bruxism. [18–20]. Despite the multifactorial etiology of bruxism which precludes a single standard treatment, the stabilization splint is considered the gold standard treatment method in the literature [21] However, depending on the etiology of bruxism, one or more of different treatment methods such as pharmacotherapy, physiotherapy, behavioral methods, botulinum toxin injection, as well as stabilization splints can be preferred for each patient. [18, 20, 22]. In the diagnosis of bruxism, patient self-reporting, clinical examination, electromyography, and polysomnography (PSG) are used. PSG is accepted as the gold standard for the diagnosis of bruxism, and it also provides the opportunity to evaluate sleep disorders, especially OSA, which are among the etiological factors of bruxism [18, 23].

As can be seen, the etiology, treatment options, and diagnostic methods of bruxism are quite varied and complex. This makes it difficult to provide accurate and comprehensive information about bruxism treatment to non-health professional users on an internet platform like YouTube. Motivated by this concern, we aimed to evaluate the content and quality of YouTube videos on bruxism treatments, a topic that has not been previously covered in the literature to our knowledge. Our working hypothesis is that a significant portion of YouTube videos on bruxism treatments are of low quality and have insufficient information, and are therefore potentially misleading to non-professional users seeking health information online.

## Methods

### Youtube Search

We used Google Trends to determine the most appropriate search term to reveal the videos that constitute the population of our study. Google Trends is an online search tool that highlights the most frequently utilized keywords and phrases within a specific timeframe. Following a search on Google Trends for this purpose, it was established that the most commonly used keywords pertaining to bruxism treatments were ‘bruxism treatment’ and ‘teeth grinding treatment,’ with both terms being searched approximately equally. For this reason, it was preferred to use both search terms in the study.

Subsequently, on December 19, 2023, a YouTube search was conducted using the keywords “bruxism treatment” between 13:00 and 16:00 and “teeth grinding treatment” between 17:00 and 20:00. The “sort by relevance” filter was used for both search terms and the first 150 videos were recorded.

Videos that were in English and had acceptable video and audio quality were included in the study. Those videos produced in languages other than English, those lacking written or audio explanations, conference lectures, duplicate content, videos surpassing 30 min in duration, and those unrelated to bruxism treatment were excluded from the study.

### Variables

The videos were evaluated separately by three researchers by recording the following variables. All reviewers were blinded to each other’s responses.

The number of views (1), video duration (second) (2), likes (3), dislikes (4), number of comments (5) and upload date (6) were recorded. Using this data, the viewing rate (7) (number of views/number of days since upload X 100%) and interaction index (8) ([number of likes - number of dislikes]/Total number of views X 100%) were calculated. The sources that uploaded the video were categorized as university-hospital-institute (1), dentist (2), medical doctor (3), physiotherapist (4), healthcare company (5), individual (6) or other (7). Video type was classified as educational (1), patient experience (2). The audience targeted to be reached with the video was categorized as professional (1), patient (2) and both (3).

The types of treatments recommended or introduced for the treatment of bruxism (splint, botulinum toxin injection, physiotherapy, complementary medicine practices, lifestyle changes, behavioral methods, psychotherapy, recommendations (heat application, etc.), medical treatment and dental rehabilitation were recorded.

The video content was evaluated based on whether it covered nine different items, including the definition of the disease (1), subtypes of the disease (2), etiology (3), description of relevant treatment (4), various treatment modalities (5), indications/contraindications (6), complications (7), clinical findings (8), and prognosis (9). Each item was given a score of 1, thus the videos were rated with a total usefulness score ranging from 0 to 9. Using this score, the videos were divided into 3 groups in terms of their usefulness;

- **Poor**: Videos with a total score of 0 to 3 that are not useful for patients.
- **Moderate**: Videos with a total score of 4 to 6 that contain some useful information for patients.
- **Excellent**: Videos with a total score of 7 to 9, examining the subject from many aspects and containing detailed and useful information.

We also used the modified DISCERN tool to evaluate video quality. This tool is a simplified and shortened modification of the DISCERN tool, which was designed to assess the quality of written information about treatment options. The modified DISCERN consists of 5 questions (Table 1) positive answers are scored as 1 and negative answers are scored as 0. The tool’s potential total score is 5 points, with higher scores indicating higher video quality.

Ethics committee approval was not required for the study.

**Table 1:** Modified DISCERN (1 point is given for every yes and 0 points for no)

1. Are the aims clear and achieved?
2. Are reliable sources of information used? (i.e., publication cited, speaker is board-certified general surgeon)
3. Is the information presented balanced and unbiased?
4. Are additional sources of information listed for patient reference?
5. Are areas of uncertainty mentioned?

### Statistical methods

Statistical analysis was performed using IBM SPSS Statistical Software (version 21; IBM, Armonk, NY). While evaluating the study data, frequencies (number, percentage) were used for categorical variables, and descriptive statistics [mean, standard deviation (SD), median (IQR-interquartile range)] were used for numerical variables. Continuous variable normality was assessed using the Shapiro-Wilk test. The differences between two independent groups were analyzed using the Mann–Whitney test. Differences between more than two independent groups were analyzed using the Kruskal–Wallis test. The relationships between independent numerical variables were checked with the Spearman correlation coefficient, while the relationships between categorical variables were checked using chi-square analysis. Statistical significance in the analysis was interpreted at the level of 0.05.

## Results

The first 150 videos for each term (*n* = 300) related to bruxism were scanned. Eighty-one of the videos had the same URL address and 80 were excluded according to predetermined study criteria. The remaining 139 videos were analyzed in this study. The interobserver agreement for the usefulness score and DISCERN calculated as kappa score was 0.857, for treatment modalities calculated as kappa score 0.955.

The videos ran from 12.00 s to 1342 s (~ 22 min), with a median runtime of 184 s (~ 3 min). The number of overall views per day ranged from 0.02 to 3574.65 with a median of 4.09. The videos yielded 15,111,248 total of views with median of 4850 views. As of the data collection date, videos had been available online for 93 days to 5548 days, with a median of 1024 days. The median interaction index score was 0.91% (range from 0 to 100%; mean = 2.13 ± 8.56) and the rate of view was 409 (range from 1.59 to 357465.19%; mean = 6994.33 ± 35806.12). The descriptive statistics of the videos are presented in Table 2.

**Table 2:** Descriptive statistics

Demographics	Mean ± SS	Median (IQR)	Minimum – Maximum
Views per day	69.94 ± 358.06	4.09 (23.21)	0.02; 3574.65
Viewing rate	6994.33 ± 35806.12	409.00 (2320.85)	1.59; 357465.19
Days since upload	1473.95 ± 1167.04	1024.00 (1491.00)	93.00; 5548.00
Interaction index	2.13 ± 8.56	0.91 (1.35)	0.00; 100.00
Number of views	108714.01 ± 589747.05	4850.00 (27743.00)	18.00; 5658674.00
Video duration	258.10 ± 219.63	184.00 (234.00)	12.00; 1342.00
Likes	1380.33 ± 7468.48	36.00 (275.00)	0.00; 69000.00
Dislikes	0.01 ± 0.17	0.00 (0.00)	0.00; 2.00
Number of comments	119.99 ± 621.16	1.00 (28.00)	0.00; 6132.00
Discern	3.01 ± 0.99	3.00 (2.00)	0.00;5.00

Videos were categorized according to their source categories. Almost half of these videos (53.2%, *n* = 74) were uploaded by dentists, whereas 12.9% (*n* = 18) were uploaded by medical doctors. Additionally healthcare companies and university-hospital-institute had similar percentages of uploaded (5,8%, *n* = 8), and 4.3% (*n* = 6) of videos were uploaded by physiotherapists and similar percentages of uploaded videos were recorded with individual users (4.3%, *n* = 6), while 13.7% (*n* = 19) were uploaded by others. Videos were also classified based on their type. The great majority of videos were uploaded for informational purposes (93.5%, *n* = 130) and 6,5% (*n* = 9) were uploaded for sharing their own experience. Additionally, the most targeted audience of all videos was patients (87.1%, *n* = 121) followed by healthcare professionals (5,8%, *n* = 8), and both patients and professionals (7,1%, *n* = 10). More than half of the videos (56.2%, *n* = 76) were uploaded by users in the United States followed by Australia (10.4%, *n* = 14), England (10.4%, *n* = 14), India (10.4%, *n* = 14), Singapore (3%, *n* = 4), Spain (2.2%, *n* = 3), Turkey (1.5%, *n* = 2), Dubai (1.5%, *n* = 2), Canada (1.5%, *n* = 2) and others (3%, *n* = 4).

The YouTube videos contained variable information on bruxism, teeth clenching and/or grinding. According to the presence of information in 9 non-mutually exclusive domains, an average of 3.33 ± 1.80 domains were discussed in these videos. In total, 59% of the videos were poor (*n* = 82), 36.7% were moderate (*n* = 51) and %4.3 were found excellent (*n* = 6). The most discussed topics were procedures involved in the related treatment (76.3%, *n* = 106), clinical signs and symptoms (57.6%, *n* = 80), definition of the disorder (45.3%, *n* = 63), alternative treatment modalities (45.3%, *n* = 63) and etiology (44.6%, *n* = 62). Prognosis (31.7%, *n* = 44) and subgroups of bruxism (18.7%, *n* = 26) were described in some of the videos, whereas indication/contraindication (7.9%, *n* = 11) and complication of a treatment modality (5.8%, *n* = 8) were poorly mentioned in the evaluated videos. The descriptive statistics of the video demographic data according to the usefulness score indicating the quality of the video and the associations between them are shown in Table 3. A statistically significant difference was found between the content usefulness score and the interaction index (χ2 = 6.587, *p* = 0.037*).* Videos with moderate-quality content had a significantly higher interaction index than did those with excellent-quality content (*p* = 0.039*).* Additionally, a significant relationship was found between the content usefulness score and video length (χ2 = 12.842, *p* = 0.002). Videos with excellent-quality content were significantly longer than videos with moderate-quality (*p* = 0.024) or poor-quality content (*p* = 0.002). There were no other significant differences between the content usefulness score and the video demographic data. There were no statistically significant differences between the content usefulness score and the source of upload (*p* = 0.154), the content of the video (*p* = 0.629) or the target audience (*p* = 0.242).

**Table 3:** Comparison of descriptive data according to usefulness score

	Poor-quality (*n* = 82)	Moderate-quality (*n* = 51)	Excellent-quality (*n* = 6)	Test statistics	
	**Mean ± SS** **Median (IQR)**	**Mean ± SS** **Median (IQR)**	**Mean ± SS** **Median (IQR)**	$\:{}^{2}$	*p*
Views per day	97.17 ± 463.06	29.76 ± 58.37	39.42 ± 31.90	$\:\chi\:2$=4.065	0.131
	3.77 (20.74)	3.27 (33.92)	38.80 (56.52)		
Viewing rate	9717.01 ± 46306.08	2975.76 ± 5837.81	3942.08 ± 3190.39	$\:\chi\:2$=4.065	0.131
	377.47 (2074.09)	327.05 (3392.18)	3880.09 (5651.67)		
Day	1516.41 ± 1149.52	1473.82 ± 1242.16	894.67 ± 567.97	$\:\chi\:2$=1.198	0.549
	1204.50 (1799.00)	997.00 (1438.00)	828.50 (807.00)		
Interaction index	1.61 ± 2.17	2.98 ± 13.90	2.00 ± 0.59	$\:\chi\:2$=6.587	**0.037**
	0.99 (1.56)	0.81 (1.15)	1.83 (1.04)		
Number of views	156440.00 ± 763524.29	40518.82 ± 78276.66	36118.00 ± 53220.28	$\:\chi\:2$=1.957	0.376
	4922.00 (22003.00)	3560.00 (55915.00)	13568.50 (53611.00)		
Video duration	236.74 ± 219.64	260.49 ± 207.79	529.67 ± 148.42	$\:\chi\:2$=12.842	**0.002**
	158.00 (264.00)	202.00 (195.00)	501.50 (295.00)		
Likes	2072.04 ± 9654.77	358.47 ± 977.93	612.83 ± 795.43	$\:\chi\:2$=4.408	0.110
	41.50 (195.00)	29.00 (325.00)	340.50 (728.00)		
Dislikes	0.02 ± 0.22	0.00 ± 0.00	0.00 ± 0.00	$\:\chi\:2$=0.695	0.706
	0.00 (0.00)	0.00 (0.00)	0.00 (0.00)		
Number of comments	169.18 ± 802.29	48.31 ± 109.62	57.00 ± 68.15	$\:\chi\:2$=3.497	0.174
	1.00 (27.00)	3.00 (40.00)	29.00 (96.00)		

$\:{}^{2}$= Kruskal Wallis Test Statistics

In addition to usefulness scores the quality of the videos was also evaluated with the DISCERN form. The videos achieved a median DISCERN score of 3 (range from 1 to 5; mean = 3.06 ± 0.93). When DISCERN values were compared to content usefulness scores, videos with poor-quality content were found to have significantly lower DISCERN scores than videos with moderate-quality (*p* < 0.001) or excellent-quality content (*p* = 0.008) (Table 4). Additionaly, there was a significantly positive and moderate (*r* = 0.446) relationship between DISCERN scores and content usefulness scores (*p* < 0.001). The correlation between DISCERN score and video demographic data are demonstrated on Table 5. There was only a weak positive correlation between DISCERN scores and video length (*r* = 0.359; *p* < 0.001). No other significant correlations were found between DISCERN scores and other demographic video data. No statistically significant differences were found between DISCERN values and the target of the video (*p* = 0.969), the source of the upload (*p* = 0.293), or the content of the video (*p* = 0.835).

**Table 4:** Pairwise comparisons of DISCERN scores based on usefulness scores

Discern	*p*
Poor quality — Moderate quality	**< 0.001**
Poor quality —Excellent quality	**0.008**
Moderate quality — Excellent quality	0.506

*Bonferroni Corrected p values

**Table 5:** Relationship between Discern score and youtube characteristics

	Discern	
	*r*	*p*
Views per day	0.163	0.055
Viewing rate	0.163	0.055
Days since upload	-0.001	0.993
Interaction index	0.113	0.186
Number of views	0.156	0.067
Video duation	0.351	**< 0.001**
Likes	0.201	**0.018**
Dislikes	-0.154	0.070
Number of comments	0.192	**0.024**

r: Spearman Correlation Coefficient

The comparison of YouTube characteristics according to upload source presents on the Table 6. The videos uploaded by physiotherapists had significantly higher views per day and viewing rates than did the videos uploaded by medical doctors (*p* = 0.037), university-hospital-institute (*p* = 0.024) and dentists (*p* = 0.006). The videos uploaded by physiotherapists also had a remarkably higher total number of views than did those uploaded by university-hospital-institute (*p* = 0.041) and dentists (*p* = 0.012). The length of the videos uploaded by physiotherapists and individual users were significantly longer than those of uploaded by university-hospital-institute (*p* = 0.014; *p* = 0.009, respectively) and dentists (*p* = 0.016; *p* = 0.010, respectively). Additionally, the videos uploaded by physiotherapists and individual users showed significantly higher interaction index than those of uploaded by university-hospital-institute (*p* = 0.018 and *p* = 0.017, respectively). Additionaly, the videos uploaded by physiotherapists had a notably greater number of likes and comments than the videos uploaded by medical doctors (*p* = 0.023; *p* = 0.009, respectively), university-hospital-institute (*p* = 0.003; *p* = 0.008, respectively) and dentists (*p* = 0.002; *p* = 0.002, respectively).

**Table 6:** Comparison of Youtube characteristics by upload of source

	University-Hospital-Institute (*n* = 8)	Dentist (*n* = 74)	Medical doctor (*n* = 18)	Physiotherapist (*n* = 6)	Healtcare company (*n* = 8)		Individual (*n* = 6)	Other (*n* = 19)	
	Mean ± SS Median(IQR)	Mean ± SS Median(IQR)	Mean ± SS Median(IQR)	Mean ± SS Median(IQR)		Mean ± SS Median(IQR)	Mean ± SS Median(IQR)	Mean ± SS Median(IQR)	p
Views per day	7.55 ± 11.17	20.26 ± 41.89	15.81 ± 27.66	1182.15 ± 1393.69	17.97 ± 18.75		17.33 ± 23.46	28.27 ± 64.01	**0.012**
	1.30 (14.53)	2.51 (21.81)	3.33 (24.18)	746.15 (2348.24)	10.48 (27.76)		5.76 (36.51)	8.04 (15.80)	
Viewving rate	754.65 ± 1117.26	2026.22 ± 4189.64	1580.67 ± 2765.78	118215.08 ± 139369.40	1797.31 ± 1874.79		1733.32 ± 2346.02	2826.98 ± 6400.60	**0.012**
	130.11 (1453.40)	251.19 (2181.38)	332.95 (2418.38)	74615.02 (234823.59)	1048.13 (2776.41)		575.66 (3650.95)	803.63 (1580.41)	
Days since upload	1291.38 ± 1149.76	1494.53 ± 1204.33	1498.50 ± 1167.69	1269.00 ± 661.11	1629.00 ± 1730.27		851.33 ± 580.03	1643.47 ± 1085.04	0.801
	893.50 (1956.00)	1085.50 (1687.00)	1160.50 (1719.00)	1254.00 (1098.00)	820.50 (3311.00)		586.50 (766.00)	1519.00 (1798.00)	
Interaction index	0.38 ± 0.41	1.47 ± 2.19	1.64 ± 1.56	2.59 ± 1.56	1.07 ± 0.78		18.67 ± 39.87	0.97 ± 0.84	**0.005**
	0.29 (0.82)	0.80 (1.35)	1.02 (2.22)	2.27 (2.53)	0.93 (1.38)		2.68 (27.28)	0.91 (1.27)	
Number of views	19454.50 ± 33741.49	22664.27 ± 42825.97	18616.11 ± 34811.32	1942628.83 ± 2302154.99	21166.00 ± 24848.97		9920.00 ± 12359.44	55723.42 ± 110303.33	**0.025**
	1082.50 (43,125)	3543.50 (23,108)	4226.0 (21,510)	1335157.0 (3,852,720)	5158.0 (43,136)		5734.0 (17,830)	7319.00 (26185.00)	
Video duration	161.00 ± 89.84	247.69 ± 228.96	241.83 ± 169.22	495.50 ± 239.06	285.25 ± 211.34		523.00 ± 255.82	184.89 ± 150.16	**0.013**
	159.50 (128.00)	175.00 (237.00)	187.50 (163.00)	537.50 (422.00)	232.5 (291.00)		526.50 (409.00)	121.00 (223.00)	
Likes	67.88 ± 99.44	226.27 ± 460.32	304.39 ± 680.17	26060.67 ± 27608.35	132.75 ± 158.55		277.83 ± 502.49	526.68 ± 1489.94	**0.004**
	9.50 (145.00)	19.50 (276.00)	24.50 (256.00)	22000.00 (47943.00)	57.50 (259.00)		71.00 (394.00)	67.00 (257.00)	
Dislikes	0.00 ± 0.00	0.03 ± 0.23	0.00 ± 0.00	0.00 ± 0.00	0.00 ± 0.00		0.00 ± 0.00	0.00 ± 0.00	0.990
	0.00 (0.00)	0.00 (0.00)	0.00 (0.00)	0.00 (0.00)	0.00 (0.00)		0.00 (0.00)	0.00 (0.00)	
Number of comments	10.13 ± 16.82	29.50 ± 58.10	27.28 ± 58.44	2089.67 ± 2373.16	10.00 ± 11.14		28.33 ± 35.10	59.79 ± 153.07	**0.009**
	0.00 (23.00)	1.00 (27.00)	1.00 (24.00)	1535.00 (3897.00)	5.00 (14.00)		13.50 (65.00)	1.00 (28.00)	

$\:{}^{2}$= Kruskal Wallis Test Statistics

The videos uploaded for sharing their own experience had higher interaction index than videos uploaded for informational purposes (*p* = *0.037*). A statistically significant difference was found between the target audience and the duration of the videos (*p* = 0.008). Videos targeted toward both professionals and patients had longer durations than videos targeted toward patients (*p* = 0.014).

Among the continuous variables, Spearman’s correlation demonstrated very high positive correlations between the number of views per day and the number of views, the number of likes and the number of comments, respectively (*r* = 0.935; *p* < 0.001, *r* = 0.900; *p* < 0.001, *r* = 0.816; *p* < 0.001). There was a weak positive correlation between the number of views per day and the length of the video (*r* = 0.363; *p* < 0.001). Also, there was very weak positive correlation between the number of views per day and the interaction index of videos (*r* = 0.191; *p* = 0.025). The number of days a video had been online was negatively correlated with the interaction index (*r*=-0.467; *p* < 0.001). There was a weak positive correlation between the number of days a video has been online and the number of views (*r* = 0.366; *p* = 0.038). Weak positive correlations were found between interaction index and the length of video, the number of comments, respectively (*r* = 0.371; *p* < 0.001, *r* = 0.237; *p* = 0.005). On the other hand, there were very strong positive correlations between the number of views and the number of likes and the number of comments, respectively (*r* = 0.876; *p* < 0.001, *r* = 0.830; *p* < 0.001). Also, a weak positive correlation was recorded between the number of views and the length of the video (*r* = 0.276; *p* = 0.001). The length of the video showed a moderate positive correlation with the number of likes (*r* = 0.427; *p* < 0.001). In addition, there was a weak positive correlation between the length of the video and the number of comments (*r* = 0.376; *p* < 0.001). The number of likes showed very high positive correlations with the number of comments (*r* = 0.860; *p* < 0.001).

## Discussion

Clinicians usually encounter the false beliefs of their patients which might affect patients’ compatibility with the appropriate treatment modality [24]. Since false information regarding health problems spreads very quickly through the internet, patients might be easily misdirected to debated treatments. [25] Based on this concern, for the first time, we evaluated videos on bruxism treatments on YouTube, an open-access site frequently used to obtain health-related information [9, 26].

Most studies [9, 14, 27–30] evaluating different topics in the field of oral and maxillofacial surgery state that YouTube videos are poor-quality and therefore should not be used as a reliable source of information. Similarly 59% of the videos on YouTube about bruxism treatment had poor video quality and only 4.3% had excellent video quality. However, two different studies evaluating YouTube videos about Botox injection for the treatment of wrinkles [31] and bruxism treatment [32] have shown that the videos have excellent-quality content and can be used as a useful resource for patients. These different results can be explained by the fact that, compared with surgical procedures (orthognathic surgery, genioplasty, sinus lift, etc.), which are the subject of other studies, Botox injection focuses on a more limited area, and is less complex, therefore it is easier to convey the relevant information.

Bruxism has a complex etiology involving many factors [33], therefore there is no single standard treatment method. With a multidisciplinary approach in the management of bruxism; different treatment methods are used by professionals. [34] The findings of the current study also indicated the diversity of professions that were interested in the treatment of bruxism on YouTube. Furthermore, no statistically significant differences were noted between the video source and usefulness score groups and DISCERN scores in the present study. However, the comparison between the upload of source and YouTube data indicated striking differences. It was observed that the videos uploaded by physiotherapists have a statistically significant and dramatically higher viewing rate, number of views, number of likes and number of comments. When we re-examined the videos to find a possible explanation for this dramatic difference, it was noted that the physiotherapists had a relatively high number of followers. Due to this important difference, we thought that the high number of followers may also be effective in terms of the high viewing rate, number of likes and comments of the videos uploaded by physiotherapists. Another possible explanation for this higher viewing rate might also be that physiotherapists usually offer daily routines and/or self-exercises that can be immediately applied by patients.

The video duration and interaction index were found to be significantly higher in individual videos in the present study. Individual videos are generally videos in which patients share their treatment processes and experiences. In general, professionals share information in a planned manner while avoiding unnecessary information. On the other hand, patients mostly described their bruxism experiences, treatment processes and effects in more detail and in a conversational manner. This also explains the longer duration of individual videos. Additionally, since real-life experiences are shared in individual videos, there is a higher potential for establishing an emotional bond and developing empathy between the viewer and the narrator. This situation can be considered to have an impact on the high interaction index. A high interaction index can also be interpreted as individual videos being taken into consideration by patients. Although there was no significant difference between the individual videos and the usefulness score and DISCERN in our study, it can be speculated that this is related to the small number of individual videos (*n* = 6). According to the literature, there are studies showing that individual videos contain incorrect information and are less educational [28, 35, 36].

In our study, the majority of the videos (87.6%) were aimed at informing patients and a small portion (6.2%) were aimed at informing professionals. In another study evaluating YouTube videos about arthrocentesis [9], 37.2% of the videos were for professionals and 20.9% were for patients. The reason for this difference may be that the treatment of bruxism includes suggestions such as lifestyle changes, behavioral methods and hot massage that are noninvasive and that patients can apply themselves, and videos have been prepared to share these suggestions with patients. In fact, the noninvasive methods mentioned above are recommended for 56.4% of the videos. However, arthrocentesis is a specific and invasive method, and it is more likely that surgeons will prepare videos aimed at informing professionals about this method.

The two least mentioned topics that make up the usefulness score in videos about bruxism treatment are indication-contraindication (7.9%) and complications (5.8%). However, knowing the possible complications of a treatment option is one of the important factors that greatly affects the patient’s decision to consent to that treatment. The fact that this information is low in the videos, the majority of which (82.6%) are uploaded by professionals and whose target audience is mostly patients (87.6%), may be explained by the fact that treatment options other than botulinum toxin injection in the treatment of bruxism are noninvasive methods and do not have significant complications.

However, in the literature, results consistent with our study have been reported in studies evaluating YouTube videos on invasive operations with many possible complications, such as orthognathic surgery [14], implant surgery [30], impacted tooth surgery [37], and arthrocentesis [9], revealing that there is a tendency not to include complication information. The basis of this tendency may be the concern that the patient will have a negative attitude towards the mentioned treatment option and the professional who mentioned its complications.

Our study has some limitations. First the search results on YouTube vary depending on date and time, and new videos are constantly added and deleted, therefore the evaluation can only include videos recorded at a certain moment. The second is to evaluate only English content on YouTube, which has many videos in many different languages.

## Conclusions

Most YouTube videos on bruxism treatment have poor quality content. This may cause patients to turn to treatment methods that are not suitable for them, which may cause their complaints to persist or worsen. For this reason, healthcare professionals should advise patients to be careful about such potentially misleading content and direct them to reliable content when deemed necessary.

### Electronic supplementary material

Below is the link to the electronic supplementary material.

Supplementary Material 1

## Data Availability

The datasets used and/or analysed during the current study are available from the corresponding author on reasonable request.
