# Supplementary material for: Bruxism treatment on Youtube: evaluating reliability and information accuracy
Source: BMC Oral Health. 2024 Jul 15;24:798. doi: 10.1186/s12903-024-04571-5 (PMC11250974; doi:10.1186/s12903-024-04571-5)
Supplement: Supplementary file 1 — Supplementary Material 1 [file 12903_2024_4571_MOESM1_ESM.docx]

**Appendix 1.**

**Comparison of Upload of Source, Video Type and Target Audience according to Usefulness score**

|  | **Poor quality**  **n (%)** | **Moderate quality**  **n (%)** | **Excellent quality**  **n (%)** | **Test statistics** | |
| --- | --- | --- | --- | --- | --- |
|  |  |  |  | **χ^2^** | **p*** |
| **Upload Source** |  |  |  |  |  |
| University-hospital-institute | 3 (3.7) | 5 (9.8) | 0 (0.0) | 16.885 | 0.154* |
| Dentist | 43 (52.4) | 26 (51.0) | 5 (83.3) |  |  |
| Medical doctor | 13 (15.9) | 4 (7.8) | 1 (16.7) |  |  |
| Physiotherapist | 6 (7.3) | 0 (0.0) | 0 (0.0) |  |  |
| Healthcare company | 3 (3.7) | 5 (9.8) | 0 (0.0) |  |  |
| Individual | 3 (3.7) | 3 (5.9) | 0 (0.0) |  |  |
| Other | 11 (13.3) | 8 (15.7) | 0 (0.0) |  |  |
| **Video type** |  |  |  |  |  |
| Patient experience | 6 (7.3) | 3 (5.9) | 0 (0.0) | 0.926 | 0.629* |
| Educational | 76 (92.7) | 48 (94.1) | 6 (100.0) |  |  |
| **Target Audience** |  |  |  |  |  |
| Professional | 7 (8.5) | 1 (2.0) | 0 (0.0) | 5.471 | 0.242* |
| Patient | 68 (83.0) | 48 (94.1) | 5 (83.3) |  |  |
| Both | 7 (8.5) | 2 (3.9) | 1 (16.7) |  |  |

${}^{2}$*:* *Chi Square Test Statistics *Likelihood ratio value is given.*

**Appendix 2**

**Comparison of DISCERN Scores according to Usefulness scores**

|  | **Poor quality**  **(n=82)** | **Moderate quality**  **(n=51)** | **Excellent quality**  **(n=6)** | **Test statistics** | |
| --- | --- | --- | --- | --- | --- |
|  | **Mean±SS**  **Median(IQR)** | **Mean±SS**  **Median(IQR)** | **Mean±SS**  **Median(IQR)** | ${}^{\mathbf{2}}$ | **p** |
| **Discern** | 2.72±0.88 | 3.35±0.99 | 4.00±0.89 | $\chi2$=20.465 | **<0.001** |
|  | 3.00 (1.00) | 3.00 (1.00) | 4.00 (2.00) |  |  |

${}^{2}$*=Kruskal Wallis Test Statistics*

**Appendix 3**

**Comparison of Discern Scores by Video Source, Type and Target Audience**

|  | | **DISCERN SCORE** | | **Test Statistics*** | |
| --- | --- | --- | --- | --- | --- |
|  |  | **Mean±SS** | **Median (IQR)** | $\boldsymbol{\chi}\boldsymbol{2}$ | **p** |
| **SOURCE** | University-hospital-institute | 3.12±0.64 | 3.00 (0.75) | $\chi2$=7.309 | 0.293 |
|  | Dentist | 2.90±0.95 | 3.00 (1.00) |  |  |
|  | Medical doctor | 3.00±1.03 | 3.00 (1.25) |  |  |
|  | Physiotherapist | 2.83±0.41 | 3.00 (0.25) |  |  |
|  | Healthcare company | 3.50±1.07 | 4.00 (1.75) |  |  |
|  | Individual | 2.33±1.63 | 2.50 (3.25) |  |  |
|  | Other | 3.42±0.96 | 3.00 (1.00) |  |  |
|  |  |  |  |  |  |
| **TYPE** | Patient experience | 2.89±1.27 | 3.00 (1.50) | z=0.209 | 0.835 |
|  | Educational | 3.01±0.97 | 3.00 (2.00) |  |  |
|  |  |  |  |  |  |
| **TARGET AUDIENCE** | Professional | 2.75±1.28 | 3.00 (1.50) | $\chi2$=0.063 | 0.969 |
|  | Patient | 3.02±0.97 | 3.00 (2.00) |  |  |
|  | Both | 3.10±0.99 | 3.00 (2.00) |  |  |

${}^{2}$*=Kruskal Wallis Test Statistics, z=Mann Whitney U Test Statistics*

**Appendix 4**

**Comparison of Youtube Characteristics by Video Type**

|  | **Patient experience (n=9)** | **Educational**  **(n=130)** | **Test Statistics** | |
| --- | --- | --- | --- | --- |
|  | **Mean±SS**  **Median(IQR)** | **Mean±SS**  **Median(IQR)** | **z** | **p** |
| **Views per day** | 23.56±45.87 | 73.15±369.95 | z=0.017 | 0.986 |
|  | 6.25 (23.45) | 4.04 (24.35) |  |  |
| **Viewing rate** | 2356.04±4586.98 | 7315.44±36994.80 | z=0.017 | 0.986 |
|  | 625.47±2344.54 | 403.78 (2434.60) |  |  |
| **Days since upload** | 816.00±523.37 | 1519.50±1186.48 | z=1.738 | 0.082 |
|  | 589.00 (688.00) | 1102.00 (1613.00) |  |  |
| **Interaction index** | 12.74±32.74 | 1.39±1.86 | z=2.090 | **0.037** |
|  | 1.39 (2.72) | 0.84 (1.42) |  |  |
| **Number of views** | 25105.89±60622.68 | 114502.27±609358.22 | z=0.484 | 0.629 |
|  | 5061.00 (11108.00) | 4816.50 (29107.00) |  |  |
| **Video duration** | 383.11±233.53 | 249.45±216.92 | z=1.742 | 0.082 |
|  | 411.00 (460.00) | 178.50 (225.00) |  |  |
| **Likes** | 343.11±847.32 | 1452.14±7716.53 | z=0.578 | 0.563 |
|  | 69.00 (75.00) | 34.50 (279.00) |  |  |
| **Dislikes** | 0.00±0.00 | 0.02±0.17 | z=0.263 | 0.792 |
|  | 0.00 (0.00) | 0.00 (0.00) |  |  |
| **Number of comments** | 28.89±42.06 | 126.30±641.89 | z=0.624 | 0.533 |
|  | 17.00 (41.00) | 1.00 (29.00) |  |  |

*z=Mann Whitney U Test Statistics*

**Appendix 5**

**Comparison of Youtube Characteristics by Target Audience**

|  | **Professional**  **(n=8)** | **Patient**  **(n=121)** | **Both**  **(n=10)** | **Test Statistics** | |
| --- | --- | --- | --- | --- | --- |
|  | **Mean±SS**  **Median(IQR)** | **Mean±SS**  **Median(IQR)** | **Mean±SS**  **Median(IQR)** | ${}^{\mathbf{2}}$ | **p** |
| **Views per day** | 8.40±13.19 | 49.00±210.13 | 372.55±1125.17 | $\chi2$=3.505 | 0.173 |
|  | 1.67 (22.11) | 3.98 (23.34) | 17.91 (29.52) |  |  |
| **Viewing rate** | 840.14±1318.99 | 4900.36±21012.61 | 37254.68±112516.95 | $\chi2$=3.505 | 0.173 |
|  | 166.65 (2211.19) | 398.56 (2334.46) | 1790.89 (2951.91) |  |  |
| **Days since upload** | 1420.63±1265.91 | 1488.46±1167.85 | 1341.00±1194.78 | $\chi2$=0.374 | 0.829 |
|  | 829.00 (2022.00) | 1095.00 (1535.00) | 944.00 (1580.00) |  |  |
| **Interaction index** | 1.67±1.17 | 2.21±9.17 | 1.47±0.96 | $\chi2$=2.709 | 0.258 |
|  | 1.66 (2.29) | 0.90 (1.28) | 1.29 (1.43) |  |  |
| **Number of views** | 9628.63±18537.44 | 75828.70±375327.70 | 585894.60±1782734.99 | $\chi2$=2.344 | 0.310 |
|  | 1789.50 (9489.00) | 4783.00 (30413.00) | 11406.00 (47126.00) |  |  |
| **Video duration** | 482.25±469.10 | 231.49±183.75 | 400.80±203.69 | $\chi2$=9.612 | **0.008** |
|  | 225.00 (765.00) | 170.00 (233.00) | 351.00 (349.00) |  |  |
| **Likes** | 163.63±309.65 | 1257.12±7344.14 | 3844.50±11303.59 | $\chi2$=3.183 | 0.204 |
|  | 19.00 (275.00) | 35.00 (266.00) | 103.50 (840.00) |  |  |
| **Dislikes** | 0.25±0.71 | 0.00±0.00 | 0.00±0.00 | $\chi2$=16.375 | **<0.001** |
|  | 0.00 (0.00) | 0.00 (0.00) | 0.00 (0.00) |  |  |
| **Number of comments** | 18.25±43.28 | 107.80±601.24 | 348.90±1012.65 | $\chi2$=2.334 | 0.311 |
|  | 0.50 (15.00) | 1.00 (29.00) | 12.50 (80.00) |  |  |

${}^{2}$*=Kruskal Wallis Test Statistics*
